# The emerging roles of the DDX41 protein in immunity and diseases

**DOI:** 10.1007/s13238-016-0303-4

**Published:** 2016-08-09

**Authors:** Yan Jiang, Yanping Zhu, Zhi-Jie Liu, Songying Ouyang

**Affiliations:** 1 National Laboratory of Biomacromolecules, Institute of Biophysics, Chinese Academy of Sciences, Beijing, 100101 China; 2 iHuman Institute, Shanghai Tech University, Shanghai, 201210 China; 3 Department of Microbiology, Immunology and Molecular Genetics, University of California Los Angeles, Los Angeles, CA 90095 USA

**Keywords:** DDX41, innate immunity, RNA helicases, myelodysplastic syndrome, acute myeloid leukemia

## Abstract

RNA helicases are involved in almost every aspect of RNA, from transcription to RNA decay. DExD/H-box helicases comprise the largest SF2 helicase superfamily, which are characterized by two conserved RecA-like domains. In recent years, an increasing number of unexpected functions of these proteins have been discovered. They play important roles not only in innate immune response but also in diseases like cancers and chronic hepatitis C. In this review, we summarize the recent literatures on one member of the SF2 superfamily, the DEAD-box protein DDX41. After bacterial or viral infection, DNA or cyclic-di-GMP is released to cells. After phosphorylation of Tyr414 by BTK kinase, DDX41 will act as a sensor to recognize the invaders, followed by induction of type I interferons (IFN). After the immune response, DDX41 is degraded by the E3 ligase TRIM21, using Lys9 and Lys115 of DDX41 as the ubiquitination sites. Besides the roles in innate immunity, DDX41 is also related to diseases. An increasing number of both inherited and acquired mutations in *DDX41* gene are identified from myelodysplastic syndrome and/or acute myeloid leukemia (MDS/AML) patients. The review focuses on DDX41, as well as its homolog Abstrakt in Drosophila, which is important for survival at all stages throughout the life cycle of the fly.

## **INTRODUCTION**

RNA helicases are enzymes that utilize the energy derived from NTP hydrolysis to unwind double-stranded RNA (dsRNA) molecules (Luking et al., 1998) or disrupt RNA-protein interactions (Jankowsky et al., 2001). DExD/H-box helicases comprise the largest SF2 helicase superfamily. Within the DExD/H-box family, the proteins are further classified as DEAD, DEAH, DExH, and DExD helicases based on the amino acid sequence of the conserved motif II (Fullam and Schroder, 2013). DEAD-box proteins, which are named after the strictly conserved sequence Asp-Glu-Ala-Asp (D-E-A-D), are widely found in organisms from bacteria to humans (Linder et al., 1989). They are involved in many aspects of RNA metabolism, such as transcription, pre-mRNA splicing, transport, translation, mRNA decay, and ribosome biogenesis (Rocak and Linder, 2004). The core of DEAD-box proteins consists of two tandem repeats of RecA-like domains, with motifs I, Ia, Ib, II, and III in the N-terminal domain and motifs IV, V, and VI in the C-terminal domain. The DEAD sequence is located in motif II (Caruthers and McKay, 2002). Subsequent studies identified three additional motifs that are characteristic of the DEAD-box proteins, namely the Q-motif (Cordin et al., 2004), GG motif (Schmid and Linder, 1992), and QxxR motif (Caruthers et al., 2000).

Pathogen-associated molecular patterns (PAMPs) of pathogens can be detected by cells of the innate immune response with the help of germline-encoded pattern recognition receptors (PRRs) to induce type I interferons (IFN) (Medzhitov and Janeway, 2000). In recent years it has been reported that several DExD/H-box helicases contribute to antiviral immunity, either by acting as sensors for viral nucleic acids or by facilitating downstream signaling events. The RIG-like helicases (RLHs), including RIG-I, MDA5, and LGP2 are recognized as one of the most important groups of anti-viral PRRs (Schmidt et al., 2012). Another DEAD box helicase, DDX3 is reported to act as a sensor for viral RNA in conjunction with RIG-I and MDA5. The authors proposed that DDX3 can sensitize the RLH system for dsRNA ligands at early stages of infection when levels of RIG-I are still low (Oshiumi et al., 2010). DHX9 is identified as a sensor for dsRNA in myeloid cells (Zhang et al., 2011c), and as a sensor for CpG DNA in plasmacytoid dendritic cells (pDCs) (Kim et al., 2010). DDX1 can directly bind to poly(I:C) (Zhang et al., 2011a). DDX21 and DHX36 are located downstream of DDX1. Both DDX21 and DHX36 interact with the downstream protein TIR-domain-containing-adapter-inducing interferon-β (TRIF). This suggests that DDX1 senses dsRNA and then triggers signaling via DDX21 and DHX36 to TRIF (Zhang et al., 2011a). DDX60 is proved to act in conjunction with RIG-I or MDA5 to mediate responses to viral dsRNA (Miyashita et al., 2011). Besides the involvement in antiviral immune response, DEAD-box proteins also play important roles in virus replication. DDX1 can bind to Hepatitis C virus (HCV) 3′ (+) UTR as well as its reverse complementary 5′ (−) UTR (Tingting et al., 2006), suggesting a possible role in the initiation of HCV RNA replication. DDX1 has also been reported to be important for the human immunodeficiency virus type 1 (HIV-1) replication as it binds to and serves as a cofactor of the HIV-1 Rev protein (Fang et al., 2004). DDX3 is required for HCV RNA replication, wherein the HCV core binds to the C-terminus of DDX3 (Owsianka and Patel, 1999). DDX5 was found to interact with the HCV NS5B protein, and to be important for HCV RNA replication (Goh et al., 2004). Taken together, the DExD/H-box helicases may have a much broader role in innate immunity and cancers than previously appreciated. In this review, we will focus on the functions of DDX41 and its Drosophila homolog Abstrakt (Abs).

## **FUNCTIONS OF DDX41**

### **Functions of DDX41 in innate immunity**

**Figure 1:**
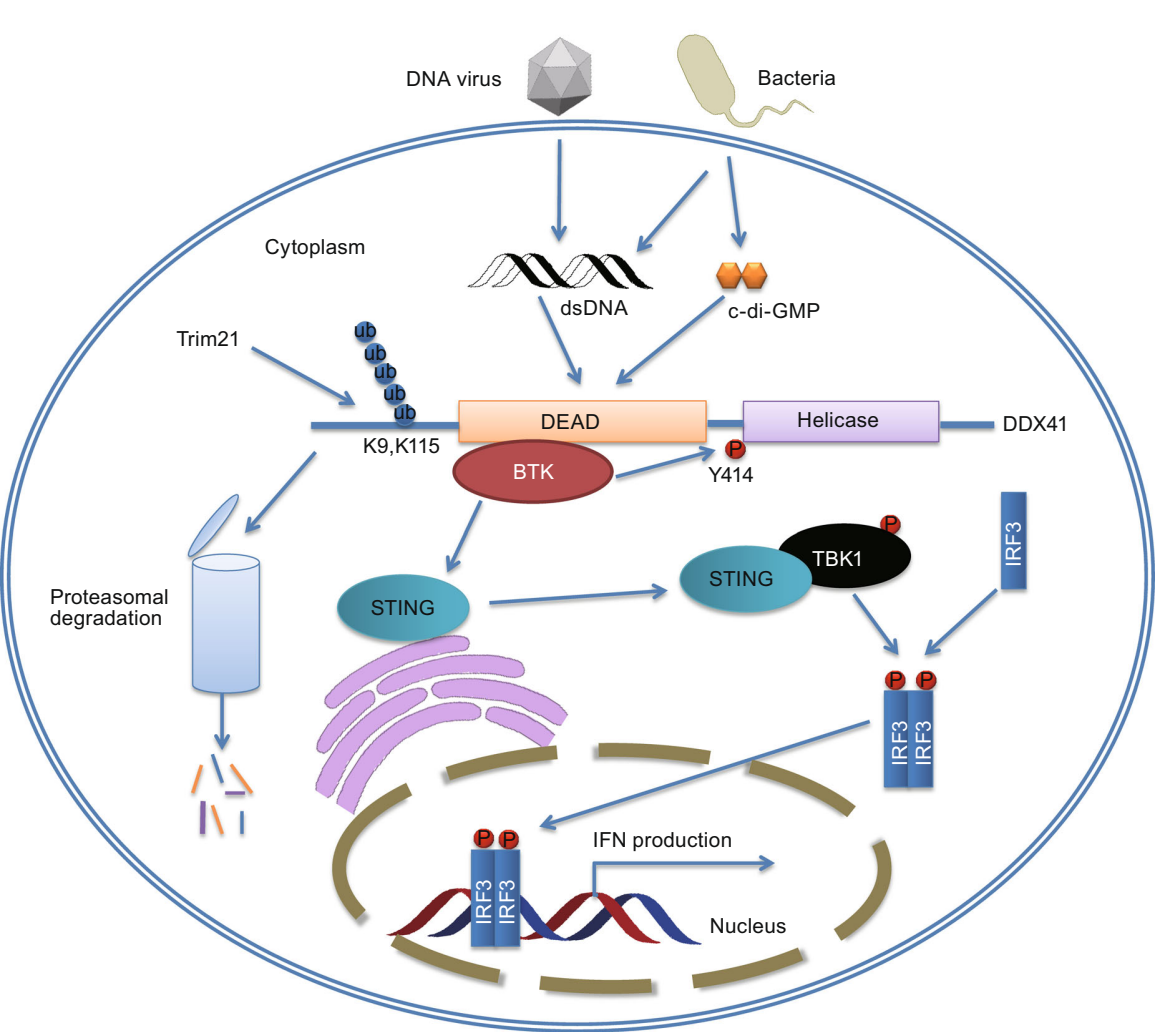
**The signaling pathway of DDX41 in innate immunity**. After infection, virus and bacteria release dsDNA or c-di-GMP to cells. DDX41 is then phosphorylated by BTK kinase at Y414 site and activated. The activated DDX41 can detect foreign PAMPs with DEAD domain, and then activate STING. Activated STING translocates from the endoplasmic reticulum (ER) to Golgi apparatus and interacts with TBK1 (Ouyang et al., 2012). The STING-TBK1 complex is required for the activation of TBK1 and the subsequent phosphorylation and nuclear translocation of IRF3, ultimately leading to expression of type I interferons. After immune response, DDX41 is ubiquitinated by Trim 21 at Lys9 and Lys115 sites, leading to degradation in proteasome

Yong-Jun Liu’s group carried out an siRNA screen with 59 members of the DExD/H-box helicase family to address potential involvement of other DExD/H-box helicases in innate immunity (Zhang et al., 2011b). They identified DDX41, a member of the DEAD-box proteins, as an intracellular DNA sensor in myeloid dendritic cells. Knockdown of DDX41 expression blocked activation of the mitogen-activated protein kinase TBK1 (TANK-binding kinase 1) and the transcription factors NF-κB and IRF3 (interferon regulatory factor) by B-form DNA but not RNA. The stimulator of interferon genes (STING) functions as a key scaffolding and adaptor protein to facilitate the signal transduction initiated from upstream cytosolic dsDNA receptors to downstream effectors TBK1 and IRF3, leading to the expression of type I IFNs (Liu and Wang, 2016). DDX41 functions through the STING-TBK1-IRF3 pathway. It directly binds DNA and STING via its DEAD domain (Fig. 1). The authors also showed that the Walker A and B motifs are required for the binding (Zhang et al., 2011b). During bacterial infection, certain bacterial species can release secondary messengers cyclic di-GMP (c-di-GMP) or cyclic di-AMP (c-di-AMP) to act as PAMP to trigger host type I interferon innate immune response (Hengge, 2009). In 2011, STING was reported to be an innate immune sensor of cyclic dinucleotides (Burdette et al., 2011). In 2012, our group determined the crystal structure of STING C-terminal domain complexed with c-di-GMP in a unique binding mode (Ouyang et al., 2012). Interestingly, Parvatiyar et al. showed that DDX41 specifically and directly interacts with c-di-GMP. Knockdown of DDX41 via short hairpin RNA inhibited immune response to c-di-GMP and resulted in defective activation of the STING-TBK1-IRF3 pathway. They proposed a three molecule synergistic binding mechanism wherein the detection of c-di-GMP by DDX41 enhances the DDX41-STING interaction, which in turn increases the binding affinity of STING and c-di-GMP; the molecular interactions eventually lead to activating the interferon response. They also demonstrated that the DEAD domain plays key roles in DDX41 detecting c-di-GMP (Fig. 1) (Parvatiyar et al., 2012). Uncontrolled sensing of DNA or RNA and excessive production of type I interferon could induce autoimmune diseases, so DDX41 must be degraded or inactivated after immune response. In 2013, Yong-Jun Liu’s group identified the E3 ligase TRIM21 as a DDX41-interacting protein. Overexpression of TRIM21 results in more degradation of DDX41 and less production of type I interferon in response to intracellular dsDNA. The SPRY-PRY domain of TRIM21 interacts with the DEAD domain of DDX41. Lys9 and Lys115 of DDX41 are the ubiquitination sites of TRIM21 (Fig. 1) (Zhang et al., 2013). However, how DDX41 functions as a DNA sensor is not completely understood. Reports in literature about DDX41 appeared very infrequently after that until 2015, when Lee et al. demonstrated that BTK-deficient cells have impaired IFN-β production and TBK1/IRF3 activation when stimulated with pathogens. Tyr364 and Tyr414 of DDX41 are critical for its recognition of DNA and binding to STING. Tyr414 is identified as the BTK phosphorylation site (Fig. 1). Besides, BTK’s kinase domain can bind the DEAD domain of DDX41 (Lee et al., 2015). Recently, the porcine DDX41 is reported involved in the dsDNA- and dsDNA-virus-mediated type I IFN signaling pathway in porcine kidney cells (Zhu et al., 2014). In addition, the function of DDX41 in innate immune regulation was also studied in teleosts. Quynh et al. cloned and characterized the DDX41-encoding gene from the olive flounder and found that olive flounder DDX41 is also a cytosolic viral DNA sensor. It showed antiviral function similar to DDX41 in mammals (Quynh et al., 2015).

### **Functions of DDX41 in human diseases**

**Figure 2:**
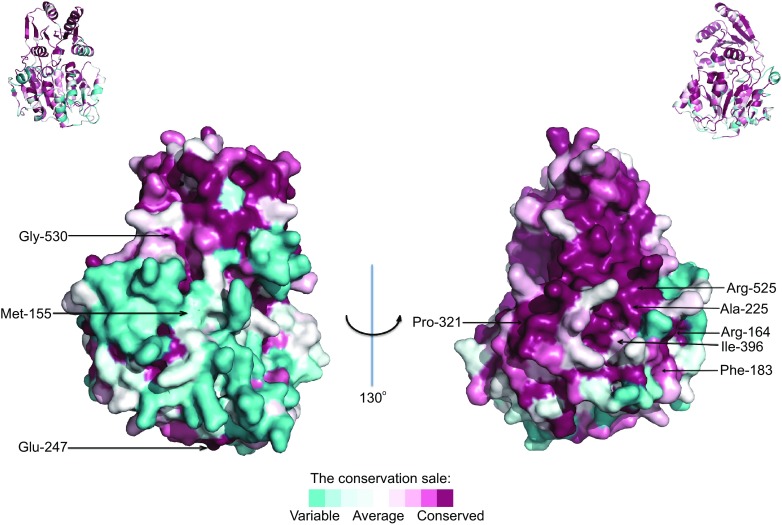
**The amino acids conservation of human DDX41 DEAD and helicase domains**. Graphical representation of the amino acids conservation using the predicted structure of human DDX41 is shown. Mutations related with MDS/AML are labeled. The color scale is based upon the level of conservation as determined by the ConSurf server. Category 9 corresponds to fully conserved residues. Small ribbon diagrams are shown at the top

Besides the roles in innate immunity, DDX41 is also related to diseases. So far, 10 genes (RUNX1, CEBPA, TERC, TERT, GATA2, SRP72, ANKRD26, ACD, ETV6, and DDX41) germline heterozygous mutations have been reported associate with familial myelodysplasia syndrome (MDS)/acute myeloid leukemia (AML) (Cardoso et al., 2016). In 2012, Somatic DDX41 mutations were reported in the study of sporadic acute myeloid leukemia (AML) syndrome by Ding et al. (2012). In 2015, Polprasert et al. identified a familial AML syndrome characterized by long latency and germline mutations in *DDX41* gene. Most of these mutations are located in DEAD domain, suggesting importance of this domain. In xenograft experiments with cell lines in which DDX41 was knocked down, they observed accelerated tumor growth compared to mock transduced cells, implying DDX41 functioning as a tumor suppressor. To further elucidate the roles of DDX41, they performed a pull down assay in HEK293 cells to identify proteins associated with DDX41. Their finding indicates that spliceosomal proteins constitute the top functional group associated with DDX41 and mutations in DDX41 (R525H) alter the native DDX41 interactome especially for major components in the U2 and U5 spliceosomes. It was concluded that DDX41 defects lead to loss of tumor suppressor function due to altered pre-mRNA splicing and RNA processing (Polprasert et al., 2015). The R525H mutation not only causes a defect in pre-rRNA processing but also inhibits cell cycle progression through the MDM2-RB-E2F axis (Kadono et al., 2016). Lewinsohn et al. identified five novel mutations including missense mutations within important functional domains, and splicing mutations predicted to result in truncated proteins from a family with MDS/AML (Lewinsohn et al., 2016). Two novel articles about germline DDX41 mutations and variants in families with MDS/AML are also published recently (Cardoso et al., 2016; Li et al., 2016). We made a structure prediction for the human DDX41 (aa 150–564) by I-TASSER server (Roy et al., 2010; Roy et al., 2012; Zhang, 2008) and analyzed amino acid conservation by the ConSurf Server (http://consurf.tau.ac.il/). We found that mutations related to diseases are conserved except M155I mutation (Fig. 2). With an increasing number of both inherited and acquired mutations in *DDX41* gene being identified, further study of how DDX41 mutations lead to hematologic malignancies is needed. Thus, DDX41 and other members of the DEAD/H-box family gene mutations are identified functionally relevant as a novel family of mutations with possible implications for prognosis and treatment of myeloid malignancies, which may lead to approaches of therapeutic schedule (Antony-Debre and Steidl, 2015).

## **FUNCTIONS OF ABSTRAKT**

Abstrakt in Drosophila is the homolog of DDX41, which shares 66% identity and 80% similarity at the amino acid level. The gene was initially identified by a very specific phenotype, the failure of the Bolwig nerve to fasciculate and project normally (Schmucker et al., 1997). Abstrakt is essential for survival at all stages throughout the life cycle of the fly. Mutants show specific defects in many developmental processes, including cell-shape changes, localization of RNA, and apoptosis. Mutations of E236K and V426M show a temperature-sensitive behavior, leading to lethality at 29°C and above (Irion and Leptin, 1999). In 2004, Irion et al. found that Abstrakt has a role in controlling cell polarity and asymmetric cell division in multiple cell types. The Abs protein interacts with *insc* RNA. Abs mutants show loss of Insc protein levels but no change of *insc* RNA levels. Although Abs is predicted to participate in RNA metabolism, they demonstrated a novel role for Abs in the post-transcriptional regulation of *insc* expression (Irion et al., 2004). An unexpected interaction of Abs and Sorting Nexin-2 (SNX2) was found by Johnny K. Ngsee’ group in 2005 (Abdul-Ghani et al., 2005). They found that the N-terminal domain of Abs interacts with the phox homology (PX) domain of SNX2, suggesting that Abs also participates in protein sorting. They also found that Abs might shuttle between the nucleus and cytoplasm with a bias towards the nucleus, and the N-terminal domain is responsible for its nuclear localization. Deletion of this domain in Abs (aa 194–622) resulted in distinct punctate cytoplasmic distribution and loss of nuclear localization (Abdul-Ghani et al., 2005).

## **CONCLUSIONS**

**Figure 3:**
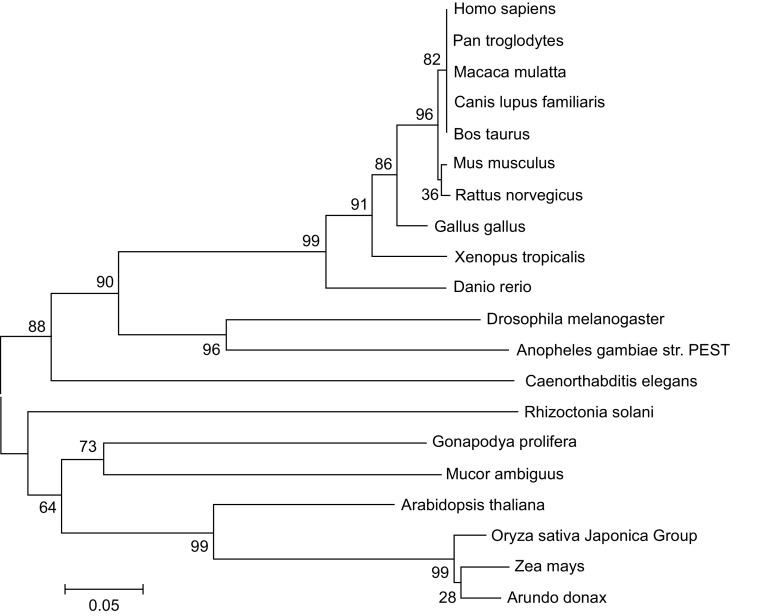
**The phylogenetic tree of DDX41 among different species**. The sequences of indicated DDX41 proteins from different species are downloaded from GenBank. The tree is generated using MEGA5 software (http://www.megasoftware.net/) with Neighbor Joining method. Length of branch represents divergence and numbers on branches are indicated as percentage of bootstrap values in 1000 sampling replicates

An increasing number of important roles of DExD/H-box helicases are discovered. In addition to the involvement in RNA metabolism, they are related to immune response, viral replication, and cell growth. The studies discussed in this review illustrate the progress being made in our understanding of the emerging roles of DDX41. DDX41 is widely distributed in eukaryotes, especially in animals. The phylogenetic tree shows that the amino acid sequence is conserved among species (Fig. 3). Although several papers showed that DDX41 is important for innate immunity, the mechanism of how it functions is still unknown. Questions remain, for example, how can it distinguish self-DNA and foreign-DNA? Furthermore, structural investigation of the DEAD domain is urgent to address a few important questions. First, is there structural difference between different DEAD domains as amino acid sequences are very similar? Second, there is a long disordered region at N-terminal except the DEAD domain and Helicase domain. Until now there has been no reports about the roles of this long disordered region. In Drosophila, the N-terminal region of the DEAD-box protein Abstrakt mediates its localization into nucleus. Is this the function of DDX41 as well? Third, Mutations in *DDX41* gene have been identified from MDS/AML patients. DDX41 defects lead to loss of tumor suppressor function due to altered pre-mRNA splicing and RNA processing. Can DDX41 be a drug target for the diseases?

In conclusion, although many new roles of DDX41 have been discovered, there are still many problems to be solved, where structural elucidation can have a significant impact. Added importance of structural investigation comes from the indication that DDX41 may function as a tumor suppressor, and could be a good therapeutic target for disease treatment.

## **ACKNOWLEDGMENTS**

This work was supported by the National Basic Research Program (973 Program) (No. 2014CB910400 and 2013CB911103), the National Natural Science Foundation of China (Grants No. 31570875, 31330019, 81590761, 31560727 and 81501353), the Beijing Nova Program (Grant No. Z141102001814020) to S.O., Youth Innovation Promotion Association CAS (Grant No. 2013065) to S.O., and the special project of Ebola virus research from the president foundation of Chinese Academy of Sciences.

## **ABBREVIATIONS**

MDS, myelodysplastic syndrome; AML, acute myeloid leukemia; BTK, Bruton’s tyrosine kinase; D-E-A-D, Asp-Glu-Ala-Asp; HCV, hepatitis C virus; IFN, interferon; IRF3, interferon regulatory factor 3; mDC, myeloid dendritic cells; PAMP, pathogen-associated molecular pattern; PRR, pattern recognition receptor; STING, stimulator of interferon genes; TBK1, TANK-binding kinase 1; TRIF, TIR-domain-containing-adapter-inducing interferon-β

## **COMPLIANCE WITH ETHICS GUIDELINES**

Yan Jiang, Zhi-Jie Liu, and Songying Ouyang declare that they have no conflict of interest. This article does not contain any studies with human or animal subjects performed by the any of the authors.
